# Prediagnostic serum glucose and lipids in relation to survival in breast cancer patients: a competing risk analysis

**DOI:** 10.1186/s12885-015-1928-z

**Published:** 2015-11-17

**Authors:** Wahyu Wulaningsih, Mariam Vahdaninia, Mark Rowley, Lars Holmberg, Hans Garmo, Håkan Malmstrom, Mats Lambe, Niklas Hammar, Göran Walldius, Ingmar Jungner, Anthonius C. Coolen, Mieke Van Hemelrijck

**Affiliations:** Cancer Epidemiology Group, Division of Cancer Studies, King’s College London, London, UK; Institute for Mathematical and Molecular Biomedicine, King’s College London, London, UK; Department of Surgical Sciences, Uppsala University Hospital, Uppsala, Sweden; Regional Cancer Centre, Uppsala, Sweden; Department of Epidemiology, Institute of Environmental Medicine, Karolinska Institutet, Stockholm, Sweden; Department of Medical Epidemiology and Biostatistics, Karolinska Institutet, Stockholm, Sweden; AstraZeneca Sverige, Södertalje, Sweden; Department of Cardiovascular Epidemiology, Institute of Environmental Medicine, Karolinska Institutet, Stockholm, Sweden; Department of Medicine, Clinical Epidemiological Unit, Karolinska Institutet and CALAB Research, Stockholm, Sweden

**Keywords:** Breast cancer, Glucose, Lipid, Competing risk, Survival, Latent class

## Abstract

**Background:**

Abnormal glucose and lipids levels may impact survival after breast cancer (BC) diagnosis, but their association to other causes of mortality such as cardiovascular (CV) disease may result in a competing risk problem.

**Methods:**

We assessed serum glucose, triglycerides (TG) and total cholesterol (TC) measured prospectively 3 months to 3 years before diagnosis in 1798 Swedish women diagnosed with any type of BC between 1985 and 1999. In addition to using Cox regression, we employed latent class proportional hazards models to capture any heterogeneity of associations between these markers and BC death. The latter method was extended to include the primary outcome (BC death) and competing outcomes (CV death and death from other causes), allowing latent class-specific hazard estimation for cause-specific deaths.

**Results:**

A lack of association between prediagnostic glucose, TG or TC with BC death was observed with Cox regression. With latent class proportional hazards model, two latent classes (Class I and II) were suggested. Class I, comprising the majority (81.5 %) of BC patients, had an increased risk of BC death following higher TG levels (HR: 1.87, 95 % CI: 1.01–3.45 for every log TG increase). Lower overall survival was observed in Class II, but no association for BC death was found. On the other hand, TC positively corresponded to CV death in Class II, and similarly, glucose to death from other causes.

**Conclusion:**

Addressing cohort heterogeneity in relation to BC survival is important in understanding the relationship between metabolic markers and cause-specific death in presence of competing outcomes.

**Electronic supplementary material:**

The online version of this article (doi:10.1186/s12885-015-1928-z) contains supplementary material, which is available to authorized users.

## Background

Disorders in glucose and lipid metabolism have been suggested as a mechanism linking obesity and breast cancer (BC) [1, 2]. In addition to their roles in carcinogenesis, increasing evidence suggests that abnormal levels of serum glucose and lipids impact survival in BC patients [3–5]. Most of these studies investigated all-cause mortality as the outcome of interest. When BC-specific death is studied as the primary outcome, information on other causes of death such as cardiovascular (CV) disease is rarely addressed in the analysis [4]. Given the high survivorship of BC [6, 7] and how glucose and lipids are linked to CV mortality [8, 9], one must consider the possibility of competing risks. For instance, a competing risk situation arises when a person has a common risk factor of dying from both BC and CV disease (and other causes), so that any earlier outcome will ‘prevent’ the individual from developing others [10]. Interpreting survival data thus becomes difficult because commonly used methods, i.e., Kaplan-Meier survival estimates and Cox’ proportional hazards, rely on the assumption of non-informative censoring. When this assumption is met, any censoring due to non-primary events does not affect one’s risk of developing the primary outcome, thus such a risk is proportional to the levels of risk factors or covariates observed. However, when competing risks are an issue a heterogeneous association between covariates and the primary outcome may exist, reflecting subpopulations or classes with different mortality risk profiles. This heterogeneity within a cohort is scarcely studied in the context of cancer survival.

The objectives of the present study were to investigate how prediagnostic serum glucose, triglycerides (TG) and total cholesterol (TC) are associated to BC death, and to capture heterogeneity of associations between these markers and BC death which may indicate a competing risk situation. We used prospectively collected data from the Apolipoprotein Mortality Risk (AMORIS) Study and utilised 1) Cox proportional hazards model to assess the link between serum glucose, TG and TC with BC death, and 2) latent class proportional hazards models with BC death as the primary outcome and deaths from CV disease and other causes as non-primary outcomes to capture heterogeneity of BC mortality risk.

## Methods

### Study population

The Apolipoprotein Mortality Risk (AMORIS) Study has been described in detail elsewhere [11, 12]. Briefly, the recently updated AMORIS database comprises 812,073 individuals with blood samples sent for laboratory testing to the Central Automation Laboratory (CALAB) in Stockholm, Sweden. Individuals recruited were mainly from the greater Stockholm area, and either healthy and having laboratory testing as a part of general check-up, or outpatients referred for laboratory testing. None of the participants were inpatients at the time the samples were analysed. In the AMORIS study, the CALAB database was linked to Swedish national registries such as the Swedish National Cancer Register, the Hospital Discharge Register, the Cause of Death Register, the consecutive Swedish Censuses during 1970–1990, and the National Register of Emigration using the Swedish 10-digit personal identity number, providing complete follow-up information until 31 December 2011.

From the AMORIS population, we selected 1798 women with an incident diagnosis of BC between 1985 and 1999 who had baseline measurements of serum glucose, TG and TC within 3 months to 3 years prior to diagnosis. Diagnosis of BC was obtained from the Swedish National Cancer Register using the Seventh Revision of the International Classification of Diseases code (ICD-7 code: 174), and information on cause-specific deaths (BC death, CV death) was obtained from the Swedish Cause of Death Register. Follow-up time was defined as the time from diagnosis until death from any causes, emigration, or end of study (31 December 2011), whichever occurred first. The ethics review board of the Karolinska Institute approved the study, and permits were obtained from Swedish Data Inspection to correlate laboratory results with Swedish national registers. Anonymity of participants was maintained throughout the study. Participant informed consent was not required for this register linkage study [13].

### Serum glucose and lipids measurements

Serum levels of glucose (mmol/L), TG (mmol/L), and TC (mmol/L) were measured enzymatically with standard methods [12]. All three markers were measured at the same day, within 3 months to 3 years prior to diagnosis. This timeframe was selected to capture metabolic derangements during ongoing malignancy process while excluding effects of breast cancer diagnostic or treatment interventions. All measurements were fully automated with automatic calibration and performed at one accredited laboratory [11]. TG levels were not normally distributed, and therefore we used log-transformed values of all markers in addition to their quartiles in the analysis.

### Covariates

Information on fasting status at baseline measurements (fasting, non-fasting, unknown) was obtained from the CALAB database. Socioeconomic status (SES; white collar, blue collar, unemployed or unknown) was based on occupational groups in the Population and Housing Census and classified all gainfully employed subjects as manual workers and non-manual workers, which were referred to as blue collar and white collar workers, respectively [14].

### Statistical analysis

We began by employing multivariable Cox proportional hazards regression to assess the association between log-transformed values and quartiles of glucose, TG and TC and the risk of BC death as the primary outcome, CV death and other death as competing outcomes. Adjustment was performed for potential confounders including age at diagnosis, SES, and fasting status at baseline measurements. Glucose, TG and TC were each analysed while adjusting for the other two markers as continuous variables. The proportionality of hazards assumption was met after assessing time-varying covariates which were the cross-products of each variable and time. To assess any potential competing risk, we used cumulative incidence functions to display the proportions of deaths from BC, CV disease and other causes by quartiles of glucose, TG, and TC.

We further investigated the association between serum glucose, TG and TC and BC survival using a latent class proportional hazards model. Latent class analysis has been used to identify different classes or latent variables within a given population which underlies the pattern of association between observed covariates [15]. In medical research, the latent class variable has been incorporated into various regression analyses, including Cox proportional hazards models, to allow identification of subgroups with different risk profiles [16–18]. To capture heterogeneity in the context of BC survival, we extended the proportional hazards model to encompass the latent class variable in addition to glucose, TG and TC, which were assessed as continuous variables. The number of latent classes present in the cohort was identified with Bayesian model selection. To assess BC-specific death whilst accounting for competing risks, we incorporated BC death as the primary outcome and deaths from CV disease and other causes as non-primary outcomes into the latent class proportional hazards model. Class membership probabilities were retrospectively predicted based on associations between covariates and events. Independent samples *T*-test and Chi^2^ test were used to assess differences in characteristics of study participants by predicted class membership. We further displayed latent class-specific cumulative incidence functions for BC, CV and other death by quartiles of the three markers. Finally, hazard ratios for BC, CV and other death by levels of glucose, TG, and TC were estimated for each latent class according to the maximum-a-posteriori (MAP) likelihood, which took into account all three outcomes [19]. More details on the latent class survival analysis are available as Additional file 1.

Descriptive analysis and Cox proportional hazards model were performed with Statistical Analysis Software (SAS) release 9.3 (SAS Institute, Cary, NC) and R version 3.0.2 (R Project for Statistical Computing, Vienna, Austria). Latent class proportional hazards model were performed with Advanced Survival Analysis software version 0.2.16 (A.C.C. Coolen, M. Rowley, M. Inoue, London, UK).

## Results

At the end of follow up (mean: 13 years), a total of 861 (47.9 %) study participants were deceased. Among these women, 425 died from BC, 179 from CV disease, and 257 from other causes. The mean age of all participants was 58 at BC diagnosis. Levels of glucose, TG, and TC were highest in those dying from CV disease, whereas women who died from BC had lower levels of the three markers compared to all women dying during follow-up period (Table 1).

**Table 1:** Descriptive characteristics of study participants overall and by causes of death

	All BC		Overall death		BC death		CV death		Other death	
	(*n* = 1798)		(*n* = 861)		(*n* = 425)		(*n* = 179)		(*n* = 257)	
	No.	%	No.	%	No.	%	No.	%	No.	%
Age, years										
Mean	58.1		62.4		56.5		71		66.2	
SD	11.8		13.2		12.5		10.3		11.4	
Follow-up time, years										
Mean	13.3		8.3		6.4		9.3		10.6	
SD	6.9		5.9		5.0		6.5		6.0	
Interval between measurements and diagnosis, months										
Mean	18.3		18.1		18.3		17.6		17.9	
SD	9.2		9.2		9.0		9.5		9.2	
SES										
White collar	648	36.0	235	27.3	147	34.6	30	16.8	58	22.6
Blue collar	894	49.7	405	47.0	222	52.2	61	34.1	122	47.5
Unemployed or unknown	256	14.3	221	25.7	56	13.2	88	49.1	77	29.9
Fasting status										
Fasting	1027	57.1	508	59.0	242	56.9	107	59.7	159	62.9
Non-fasting	568	31.6	254	29.5	133	31.3	52	29.1	69	26.8
Unknown	203	11.3	99	11.5	50	11.8	20	11.2	29	11.3
Glucose, mmol/L										
Mean	5.1		5.2		5.0		5.5		5.4	
SD	1.2		1.4		1.0		1.2		1.8	
TG, mmol/L										
Mean	1.3		1.4		1.3		1.6		1.4	
SD	0.8		0.9		0.9		0.9		0.8	
TC, mmol/L										
Mean	5.9		6.1		5.9		6.5		6.2	
SD	1.2		0.8		1.2		1.2		1.2	

**Fig. 1:**
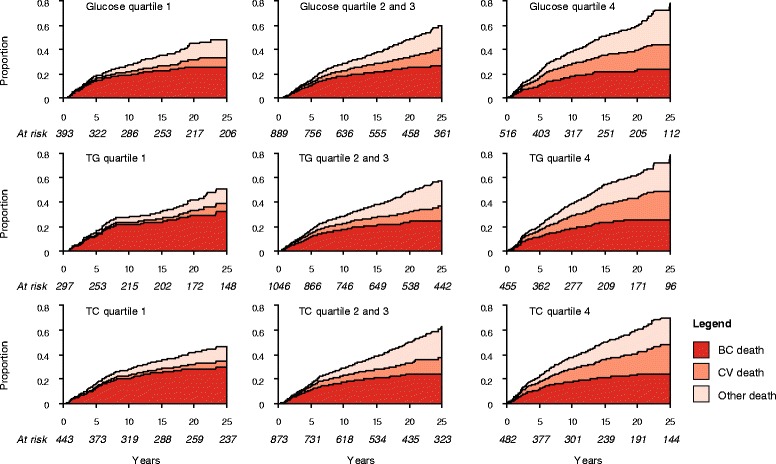
Stacked cumulative risk of death from BC, CV disease, and other causes, stratified by quartiles of glucose, TG and TC

When conventional Cox proportional hazards regression was performed, no strong association was observed between glucose, TG, and TC and risk of dying from BC (Table 2). On the other hand, positive associations were observed between TG and CV death, as well as glucose and CV death. No association was observed for other causes of death. Proportions of deaths from each causes by quartiles of glucose, TG, TC was further displayed using the cumulative incidence functions. As shown in Fig. 1, the proportion of women dying from CV disease markedly increased with higher quartiles of the markers, whilst deaths from BC are less frequent with higher quartiles of the markers. This indicated CV death as a competing event.

**Table 2:** Hazard ratios of death from BC, CV disease and other causes by levels of glucose, TG, and TC

	No. of subjects	BC death			CV death			Other death		
		No. of events	HR^a^	95 % CI	No. of events	HR^a^	95 % CI	No. of events	HR^a^	95 % CI
Glucose, mmol/L^b^										
Continuous log			0.96	0.58, 1.59		2.48	1.24, 4.96		2.09	1.16, 3.76
Quartiles										
< 4.50	393	98	1		21	1		45	1	
4.50–4.90	413	116	0.98	0.75, 1.29	36	1.27	0.74, 2.19	63	1.12	0.76, 1.64
4.90–5.30	363	96	0.95	0.72, 1.27	41	1.28	0.75, 2.19	50	0.87	0.58, 1.30
≥ 5.30	416	115	0.98	0.74, 1.29	80	1.67	1.02, 2.73	100	1.32	0.92, 1.89
*P*_trend_			0.83			0.03			0.20	
TG, mmol/L^c^										
Continuous log			1.21	0.98, 1.48		1.58	1.17, 2.13		1.32	1.02, 1.71
Quartiles										
< 0.70	297	81	1		12	1		24	1	
0.70–1.00	491	102	0.77	0.57, 1.04	34	0.91	0.46, 1.77	56	0.96	0.59, 1.57
1.00–1.60	555	132	0.97	0.72, 1.29	52	1.10	0.58, 2.08	95	1.28	0.81, 2.03
≥ 1.60	455	110	1.05	0.76, 1.45	80	1.53	0.81, 2.90	83	1.22	0.75, 1.98
*P*_trend_			0.35			0.01			0.16	
TC, mmol/L^d^										
Continuous log			0.72	0.40, 1.28		2.04	0.83, 5.04		0.67	0.32, 1.42
Quartiles										
< 5.20	443	119	1		16	1		38	1	
5.20–5.80	403	94	0.87	0.66, 1.14	37	1.52	0.83, 2.76	60	1.18	0.78, 1.79
5.80–6.60	470	102	0.79	0.60, 1.04	40	1.26	0.70, 2.27	75	1.06	0.72, 1.58
≥ 6.60	482	110	0.85	0.64, 1.15	85	1.74	0.99, 3.04	85	0.92	0.61, 1.38
*P*_trend_			0.21			0.08			0.38	

^a^Adjusted for age at diagnosis, SES (white collar, blue collar, unemployed or unknown), fasting status (fasting, non-fasting, unknown), glucose (continuous), TG (continuous), and TC (continuous)

Not adjusted for ^b^glucose, ^c^TG, ^d^TC

Our next analysis extended the proportional hazards model to include latent class variables and assess primary and non-primary outcomes. Bayesian model selection identified two latent classes in this study population. Retrospective analysis for class membership probability suggested that 81.5 % women were more likely to be members of Class I, while the other 18.5 % belonged to Class II. We further assessed baseline characteristics of study participants in relation to the most probable latent class they were assigned to. Younger average age was observed in Class I compared to Class II, and a difference in socio-economic status between classes was indicated (Table 3). With regards to clinical outcomes, no difference in proportions of women who died from BC was found between the two classes. However, statistically significantly higher overall mortality rate from CV disease and other causes were seen in Class II.

**Table 3:** Characteristics of study participants and causes of death by predicted class membership

	BC				P-value
	Class I		Class II		
	(*N* = 1466)		(*N* = 332)		
	N	%	N	%	
Age, years					<0.0001
Mean	57.6		60.5		
SD	10.9		15.0		
SES					<0.0001
White collar	554	37.8	94	28.3	
Blue collar	739	50.4	155	46.7	
Unemployed or missing	173	11.8	83	25.0	
Fasting status					0.55
Fasting	827	56.4	200	60.2	
Non-fasting	477	32.5	91	27.4	
Missing	162	11.1	41	12.4	
Glucose (mmol/l)					0.08
Mean	5.1		5		
SD	1.3		1.1		
TG (mmol/l)					0.32
Mean	1.3		1.3		
SD	0.8		0.8		
TC (mmol/l)					0.34
Mean	5.9		6.0		
SD	1.2		1.2		
BC death	342	23.3	83	25.0	0.52
CV death	129	8.8	50	15.1	<0.0001
Other death	60	4.1	197	59.3	<0.0001

**Fig. 2:**
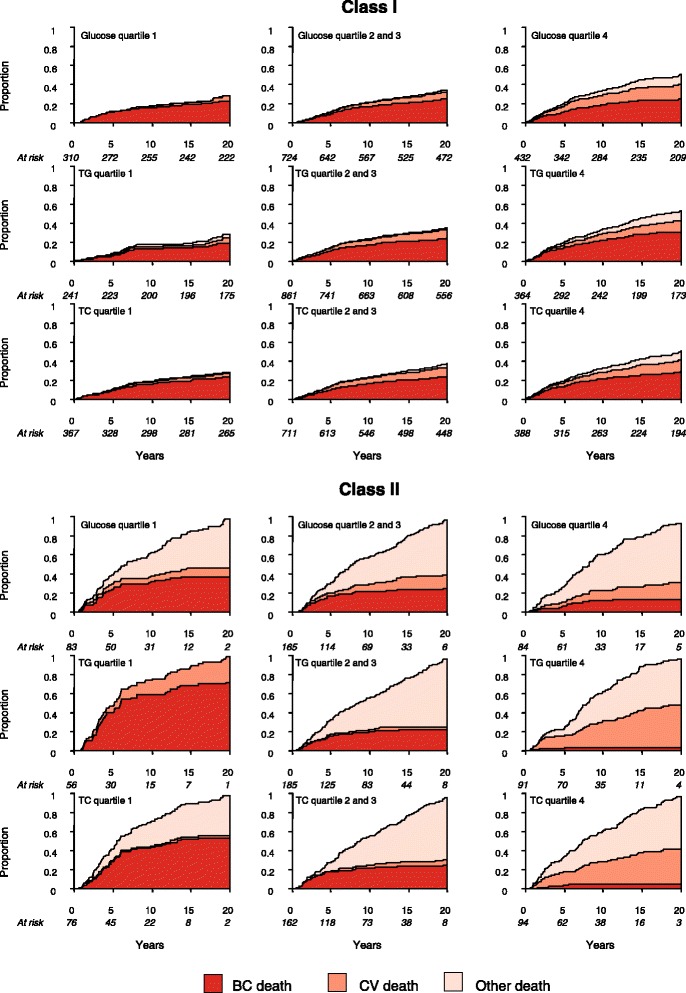
Stacked cumulative risk of death from BC, CV disease, and other causes for each latent class, stratified by quartiles of glucose, TG and TC

We further investigated difference in survivals between latent classes by displaying cumulative incidence functions for different causes of death by quartiles of glucose, TG, and TC (Fig. 2). Higher overall mortality was seen in Class II compared to Class I. In Class I, most patients died from BC, whereas in Class II, most died from other causes apart from BC and CV death. Increasing absolute numbers of deaths from BC, CV, and other causes were seen with higher levels of all three markers in Class I, although there was no marked difference in relative mortality rates between each cause of death. On the other hand, marked differences in relative proportions of women dying from the three different causes were seen across levels of markers in Class II. For instance, BC deaths were common amongst women in the lowest quartiles of glucose, TG, and TC, but contributed little to total deaths in those with highest levels of the markers. More women died from CV disease with higher TC, and a similar association was seen between glucose and death from other causes. Finally, the risk of different causes of death was quantitatively assessed by obtaining class-specific hazard estimates. As seen in Table 4, log-transformed TG corresponded to an increased risk of dying from BC in Class I, with a hazard ratio of 1.87 (95 % CI: 1.01–3.45). No statistically significant associations with BC death were observed for other markers or among women in Class II. In agreement with class-specific cumulative incidence functions, women in Class II had a higher risk of CV death with higher TC and a higher risk of other death with higher glucose levels.

**Table 4:** Hazard ratios of death from BC, CV disease and other causes by levels of glucose, TG, and TC for each latent class

	Class I		Class II	
	HR^a^	95 % CI	HR^a^	95 % CI
BC death				
Log glucose	1.09	0.73, 1.63	0.84	0.45, 1.57
Log TG	1.87	1.01, 3.45	0.91	0.50, 1.68
Log TC	0.84	0.49, 1.45	1.02	0.53, 1.99
CV death				
Log glucose	1.02	0.55, 1.91	1.46	0.97, 2.20
Log TG	7.68	2.45, 24.02	0.71	0.40, 1.25
Log TC	0.86	0.32, 2.28	2.07	1.16, 3.69
Other death				
Log glucose	0.73	0.50, 1.05	2.26	1.50, 3.40
Log TG	1.69	0.95, 3.01	1.40	0.74, 2.64
Log TC	1.20	0.65, 2.24	0.45	0.19, 1.06

^a^All covariates were included in a single model and adjusted for age at diagnosis, SES (white collar, blue collar, unemployed or unknown) and fasting status (fasting, non-fasting, unknown)

## Discussion

We performed Cox regression and a latent class proportional hazards analysis to assess the association between prediagnostic markers of glucose and lipid metabolism and death from BC in female BC patients. The latter method accounted for CV death and other death as competing risks. With the conventional Cox proportional hazards model, a lack of association was observed between the three markers and BC death. However, CV death was shown as a competing event. When latent class proportional hazards analysis were performed, we found two distinct latent classes within our cohort, reflecting different susceptibilities of dying from BC based on their baseline characteristics. Class I, comprising the majority of the study population, is associated with an increased risk of BC death following higher TG levels. Overall survival is worse in Class II, among which higher TC levels were associated with an increased risk CV death and higher glucose with risk of death from other causes. No association between the three markers and BC death was seen in Class II.

Metabolisms of glucose and lipid have been implicated in many chronic diseases. In the context of cancer, an array of evidence has linked increased BC incidence with aberrant levels of circulating glucose, TG and TC at baseline [20–22]. Abnormal levels of these markers are also associated with CV disease, which is the most common cause of death in general population [8, 9]. This has also been demonstrated in our study, as both glucose and TG were associated with a higher risk of CV death, and the associations were stronger than those with BC death. Several biological mechanisms are suggested to underlie this common link, such as chronic inflammation and insulin resistance, which may drive atherogenesis, cellular proliferation and angiogenesis [2, 23, 24]. These shared metabolic pathways may thus result in a competing risks situation, where individuals with similar sets of risk factors are equally at risk of dying from both BC and CV disease. In this case, a heterogeneous association between glucose and lipid markers and BC death may be observed, which represents subpopulations or latent classes with different mortality risk profiles. However, this heterogeneity in survival data is not addressed by common analytical methods in cancer epidemiology.

Cox proportional hazards regression and latent classes proportional hazards model differ fundamentally in the assumptions made regarding risk correlations. In Cox, non-informative censoring is assumed, which leads to the assumption of independence or no correlation between event times when multiple events are observed. However, in the real-world clinical observation, such assumptions are rarely assessable and sometimes inaccurate. The latent class proportional hazards model allows for the presence of heterogeneity underlying any observed risk associations [16] and predicts optimal parameters based on the most probable substructure of the study population. In our study, this resulted in an optimal model with two latent classes. Overall survival was lower in Class II than Class I, which indicates the importance of taking into account risk associations when investigating biological markers in relation to cancer survival.

We found TG to be associated with early death from BC in Class I. This suggests an importance of lipid metabolism in disease progression in a relevant subset of BC patients, which warrants further mechanistic investigation. No statistically significant association with BC death was observed for glucose and TC, although among Class II they were associated with higher risks of dying from other causes and CV disease, respectively. Previous studies have reported a null association for TG and TC in relation to all-cause mortality [25] and BC-specific death [26], which is similar to our findings using Cox regression and in Class II as assessed by latent classes proportional hazards model. Likewise, a lack of association with overall death has been reported for glucose [4, 5]. Although Class I comprised the majority of all women studied, it is possible that the positive association between TG and Class I was diluted in the overall cohort, resulting in a weaker association. Therefore, it is important to consider cohort heterogeneity in assessing this relationship.

The strength of this study lies in the survival analysis method used to address competing risks, as well as the relatively large cohort with follow-up information for all participants (up to 25 years). The population in the AMORIS study was selected by analysing blood samples from health check-ups in non-hospitalised persons. However, any healthy cohort effect would not affect the internal validity of our study [11]. To our knowledge, this is the first observational study utilising latent class proportional hazards model to address disease-specific survival in BC, taking into account CV death and other death as competing events. As shown in our study, the advantage of incorporating latent class analysis and multiple events in addition to proportional hazards regression is that it allows identification of subpopulations within the cohort and final survival or hazard estimates of the primary event. In other words, this method may offer a suitable approach when dealing with survival functions or hazard rates estimation in presence of competing risks. A limitation of our study was the lack of data representing older BC patients, which may partly explain the low proportion of Class II. There was no information available on tumour characteristics, BC susceptibility genes, and treatment or other metabolic and endocrine factors related to BC such as obesity and use of hormonal replacement therapy. Although residual associations with unobserved covariates were captured by our model through identification of latent classes, underlying characteristics of these different subgroups of BC patients may require further integration of other relevant markers or baseline information.

## Conclusion

The present study showed a weak association between prediagnostic TG levels and BC death in the majority of women with BC. On the other hand, glucose and TC were strongly associated to mortality from causes apart from BC in the remaining patients, among which shorter overall survival was observed. Our study therefore demonstrated heterogeneity in the association between glucose, lipid markers, and BC survival when CV death and other death were taken into account as competing outcomes. This implies an involvement of perturbed lipid metabolism in BC progression and a complex interaction between baseline biological markers and co-morbidities in determining BC survival which warrants mechanistic investigations. Therefore, our findings highlight the importance of considering cohort heterogeneity when evaluating biological markers in relation to cause-specific death.

## Additional file

Additional file 1:
**Bayesian Survival Analysis with a latent class model.** (DOC 37 kb)
