# Supplementary material for: Prediagnostic serum glucose and lipids in relation to survival in breast cancer patients: a competing risk analysis
Source: BMC Cancer. 2015 Nov 17;15:913. doi: 10.1186/s12885-015-1928-z (PMC4650114; doi:10.1186/s12885-015-1928-z)
Supplement: Additional file 1: — Bayesian Survival Analysis with a latent class model. (DOC 37 kb) [file 12885_2015_1928_MOESM1_ESM.doc]

**Additional file 1**

**Bayesian Survival Analysis with a latent class model**

**Background**

In our latent class analysis, we have developed and used a model that is able to identify a cohort sub-structure. Mathematically, such heterogeneity is incorporated in our model through,

1. the introduction of a latent class structure where *L* denotes the number of classes, and,
2. the defining of a personalised (for each patient) class and risk-specific hazard rate model having class-specific frailty, associations, and base hazard rate(s) variables.

Using such a model, our analysis is able to identify a sub-cohort structure whereby the latent classes individually obey the proportional hazards assumption, although collectively they need not; as such the analysis is able to identify and characterise cohort heterogeneity and heterogeneity-induced informative censoring and competing risks.

The extent of the permitted heterogeneity in the associations and base hazard rates across the latent classes determines the complexity of the hazard rate model. The permitted heterogeneity in the frailty and association parameters and in the base hazard rate is shown in Table S1 for the three variants of the personalised hazard rate used in our analysis. Note that regardless of whether or not the frailty, associations, and base hazard rates are heterogeneous across classes, they are always risk-specific.

**Table S1**. Complexity of model based on frailty, associations and base hazard rate

|  | **Model complexity** | | |
| --- | --- | --- | --- |
|  | **Simple (*M*=1)** | **Intermediate (*M*=2)** | **Complex (*M*=3)** |
| Frailty | Risk- and class-specific | Risk- and class-specific | Risk- and class-specific |
| Associations | Risk-specific | Risk- and class-specific | Risk- and class-specific |
| Base hazard rate | Risk-specific | Risk-specific | Risk- and class-specific |

In the implementation a parameterised form of the base hazard rate, having *K* equidistant times over the range of the survival data, is estimated using Gaussian interpolation. The complexity of the parameterised base hazard rate(s) increases with *K*.

**Implementation**

The model has been implemented in the C programming language in the Advanced Survival Analysis software version 0.2.16 (A.C.C. Coolen, M. Rowley, M. Inoue, London, UK). The task of determining the optimal parameter estimates becomes more difficult as the number of classes (*L*) is increased and the greater the complexity of the hazard rate model (*M*), and also as more complex realisations (*K*) of the base hazard rate are tested.

In determining the optimal characterisation of the cohort, Bayesian model selection (1) was been used to identify the optimal *MLK* while guarding against over-fitting. The following protocol was followed:

1. **Explore the space of latent class and hazard rate complexity for a sufficient range of classes (*L*) and hazard rate complexity (*MK*)**

The optimal frailty, associations, and base hazard rate parameters (and their error bars) are determined according to the maximum-a-posteriori (MAP) values, all estimated parameters being constrained by appropriate maximum-entropy prior. Due to the stochastic nature of the optimisation algorithm employed, this procedure is performed multiple (typically >3) times.

1. **Apply Bayesian model selection to determine the optimal number of latent classes and hazard rate model**

The model evidence for each tested latent class and hazard rate complexity combination is determined according to a Gaussian approximation to the posterior distribution. None of the tested models are given any a-priori preference.

Having determined the optimal characterisation of the cohort, “crude” and “final” (for informative censoring) survival curves are generated. Retrospective class allocation is determined using the appropriate frailty, associations, and base hazard rate(s) parameter estimates. The transformation from the parameter estimates described above to the corresponding hazard rates, 95% confidence intervals, and p-values, is also performed.

References

1. MacKay DJ. Information Theory, Inference, and Learning Algorithms. Cambridge: Cambridge University Press; 2003.
